# Tetra­methyl biphenyl-2,3,3′,4′-tetra­carboxyl­ate

**DOI:** 10.1107/S1600536808025762

**Published:** 2008-08-16

**Authors:** Guo-wei Gao, Xiao-yan Ma, Yan Jiang, Wei Chen, Jian Men

**Affiliations:** a College of Chemistry, Sichuan University, Chengdu 610064, People’s Republic of China; b College of Materials and Chemical Engineering, Chengdu University of Technology, Chengdu 610059, People’s Republic of China

## Abstract

The asymmetric unit of the title compound, C_20_H_18_O_8_, contains two mol­ecules with small geometric differences. The dihedral angles between the benzene rings are 62.94 (12) and 59.99 (12)°. The dihedral angles between the carboxylate groups in the 2- and 3-positions are 81.72 (13) and 65.54 (15)°, respectively. However, the dihedral angles between the carboxylate groups in the 3′ and 4′-positions are 67.24 (15) and 59.98 (17)°, respectively.

## Related literature

For related literature, see: Ding *et al.* (1992 ▶); Ermer (1981 ▶); Ghosh & Mittal (1996 ▶); Jiang *et al.* (2008 ▶); Rozhanskii *et al.* (2000 ▶).
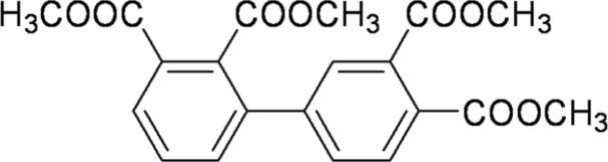


## Experimental

### 

#### Crystal data

C_20_H_18_O_8_
*M* *_r_* = 386.34
Monoclinic, 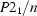

*a* = 14.395 (4) Å
*b* = 13.453 (4) Å
*c* = 21.013 (3) Å
β = 108.45 (4)°
*V* = 3860.1 (19) Å^3^
*Z* = 8
Mo *K*α radiation
μ = 0.10 mm^−1^
*T* = 297 (2) K
0.45 × 0.42 × 0.38 mm

#### Data collection

Enraf–Nonius CAD-4 diffractometer
Absorption correction: none
8168 measured reflections
6906 independent reflections
2997 reflections with *I* > 2σ(*I*)
*R*_int_ = 0.006
3 standard reflections every 300 reflections intensity decay: 1.8%

#### Refinement

*R*[*F* ^2^ > 2σ(*F* ^2^)] = 0.059
*wR*(*F* ^2^) = 0.163
*S* = 1.03
6906 reflections
515 parameters
H-atom parameters constrained
Δρ_max_ = 0.28 e Å^−3^
Δρ_min_ = −0.23 e Å^−3^

### 

Data collection: *DIFRAC* (Gabe & White, 1993 ▶); cell refinement: *DIFRAC*; data reduction: *NRCVAX* (Gabe *et al.*, 1989 ▶); program(s) used to solve structure: *SHELXS97* (Sheldrick, 2008 ▶); program(s) used to refine structure: *SHELXL97* (Sheldrick, 2008 ▶); molecular graphics: *ORTEP-3* (Farrugia, 1997 ▶); software used to prepare material for publication: *SHELXL97*.

## Supplementary Material

Crystal structure: contains datablocks global, I. DOI: 10.1107/S1600536808025762/rk2106sup1.cif

Structure factors: contains datablocks I. DOI: 10.1107/S1600536808025762/rk2106Isup2.hkl

Additional supplementary materials:  crystallographic information; 3D view; checkCIF report

## Figures and Tables

**Fig. 1.**
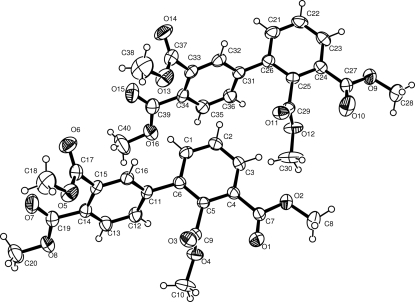
The molecular structure of I with the atom numbering scheme. Displacement ellipsoids are drawn at the 30% probability level. H atoms are presented as a small spheres of arbitrary radius.

## supplementary crystallographic information

### Comment

The title compound is a useful chemical intermediate for the further preparation of various compounds, including, for example, acid, the salts, acyl halides, amides, imides, and the like (Ding *et al.*, 1992). Polymides are well known for possessing excellent thermal and oxidative stability, as well as excellent mechanical properties (Ghosh & Mittal, 1996). The 2,3,3',4'-biphenyltetracarboxylic dianhydride was suggested as a monomer for the synthesis of soluble polyimides with high chemical and thermal stability (Rozhanskii *et al.*, 2000).

The asymmetric unit of I contains two crystallographically independent molecules (molecule 1 - C1–C18 and molecule 2 - C19–C38) (Fig. 1). For the molecule 1, the dihedral angle between the two phenyl rings are 62.94 (12)° while in the molecule 2 is 59.99 (12)° and they are both markedly differ from 42.30 (11)° in the 1,1'-biphenyl-2,3,3',4'–etracarboxylic acid monohydrate (Jiang *et al.*, 2008). For the molecule 1, the dihedral angles of the two carboxyl groups placed in 2,3-positions are 81.72 (13)° and another two carboxyl groups placed in 3',4'-positions are 67.24 (15)°; for the molecule 2, which are 65.54 (15)° and 59.98 (17)° respectively. Torsion angles C7—C4—C5—C9 and C27—C24—C25—C29 are 1.4 (4)° and 0.9 (4)°, respectively. However, torsion angles C19—C14—C15—C17 and C39—C34—C33—C37 are 7.0 (5)° and 6.2 (5)°, respectively. They are markedly smaller than those for the parent phthalic acid 20.3° (Ermer, 1981).

### Experimental

2,3,3',4'-biphenyltetracarboxylic dianhydride (29.4 g, 0.1 mol) and *p*-toluenesulfonic acid (2.0 g, 0.01 mol) was dissolved in a solution of toluene (100 ml) and methanol (50 ml) in a three-necked flask equipped with a Dean–Stark trap. The mixture was heated to reflux. After 20 h, water and most of the methanol were removed by azeotropic distillation with toluene. The mixture was allowed to cool and was added subsequently 500 ml of H_2_O. The organic phase was washed three times with saturated Na_2_CO_3_ and three times with water. Toluene was removed on a rotary evaporator and the residue was recrystallized in *Et*OH to afford white powder (34.7 g, 90% yield). Single crystals were grown by slow evaporation of a toluene of solution over a period of several days, m.p.382–384 K.

### Refinement

H atoms were positioned geometrically (C—H = 0.93–0.96Å) and refined using a riding model with the *U*_iso_(H) = 1.2*U*_eq_C (for aromatic) and *U*_iso_(H) = 1.5*U*_eq_C (for methyl).

### Crystal data

**Table:**

C_20_H_18_O_8_	*F*_000_ = 1616
*M**_r_* = 386.34	*D*_x_ = 1.330 Mg m^−^^3^
Monoclinic, *P*2_1_/*n*	Mo *K*α radiation λ = 0.71073 Å
Hall symbol: -P 2yn	Cell parameters from 31 reflections
*a* = 14.395 (4) Å	θ = 4.7–9.2º
*b* = 13.453 (4) Å	µ = 0.10 mm^−^^1^
*c* = 21.013 (3) Å	*T* = 297 (2) K
β = 108.45 (4)º	Block, colourless
*V* = 3860.1 (19) Å^3^	0.45 × 0.42 × 0.38 mm
*Z* = 8	

### Data collection

**Table:**

Enraf–Nonius CAD-4 diffractometer	*R*_int_ = 0.006
Radiation source: fine-focus sealed tube	θ_max_ = 25.3º
Monochromator: graphite	θ_min_ = 1.8º
*T* = 297(2) K	*h* = −17→16
ω/2θ scans	*k* = 0→16
Absorption correction: none	*l* = −14→25
8168 measured reflections	3 standard reflections
6906 independent reflections	every 300 reflections
2997 reflections with *I* > 2σ(*I*)	intensity decay: 1.8%

### Refinement

**Table:**

Refinement on *F*^2^	Secondary atom site location: difference Fourier map
Least-squares matrix: full	Hydrogen site location: inferred from neighbouring sites
*R*[*F*^2^ > 2σ(*F*^2^)] = 0.059	H-atom parameters constrained
*wR*(*F*^2^) = 0.163	*w* = 1/[σ^2^(*F*_o_^2^) + (0.069*P*)^2^] where *P* = (*F*_o_^2^ + 2*F*_c_^2^)/3
*S* = 1.04	(Δ/σ)_max_ < 0.001
6906 reflections	Δρ_max_ = 0.28 e Å^−^^3^
515 parameters	Δρ_min_ = −0.23 e Å^−^^3^
Primary atom site location: structure-invariant direct methods	Extinction correction: none

### Special details

**Table:**

Geometry. All s.u.'s (except the s.u. in the dihedral angle between two l.s. planes) are estimated using the full covariance matrix. The cell s.u.'s are taken into account individually in the estimation of s.u.'s in distances, angles and torsion angles; correlations between s.u.'s in cell parameters are only used when they are defined by crystal symmetry. An approximate (isotropic) treatment of cell s.u.'s is used for estimating s.u.'s involving l.s. planes.
Refinement. Refinement of *F*^2^ against ALL reflections. The weighted *R*–factor *wR* and goodness of fit *S* are based on *F*^2^, conventional *R*–factors *R* are based on *F*, with *F* set to zero for negative *F*^2^. The threshold expression of *F*^2^ > σ(*F*^2^) is used only for calculating *R*–factors(gt) *etc*. and is not relevant to the choice of reflections for refinement. *R*–factors based on *F*^2^ are statistically about twice as large as those based on *F* and *R*–factors based on ALL data will be even larger.

### Fractional atomic coordinates and isotropic or equivalent isotropic displacement parameters (Å^2^)

**Table:**

	*x*	*y*	*z*	*U*_iso_*/*U*_eq_	
O1	0.58582 (18)	0.6473 (2)	0.10659 (14)	0.0722 (8)	
O2	0.66768 (17)	0.78792 (19)	0.13706 (13)	0.0628 (7)	
O3	0.37936 (18)	0.57886 (17)	0.11045 (13)	0.0602 (7)	
O4	0.38789 (17)	0.62465 (17)	0.00901 (12)	0.0582 (7)	
O5	0.02260 (18)	0.59487 (19)	0.15264 (13)	0.0671 (8)	
O6	−0.00811 (19)	0.7556 (2)	0.16431 (13)	0.0718 (8)	
O7	−0.12482 (19)	0.6405 (2)	0.02572 (14)	0.0824 (9)	
O8	−0.08492 (18)	0.58818 (19)	−0.06318 (13)	0.0691 (8)	
C1	0.3321 (3)	0.9024 (2)	0.11080 (17)	0.0492 (9)	
H1	0.2773	0.9389	0.1110	0.059*	
C2	0.4233 (3)	0.9456 (3)	0.13244 (17)	0.0510 (9)	
H2	0.4297	1.0114	0.1467	0.061*	
C3	0.5055 (2)	0.8924 (2)	0.13322 (16)	0.0463 (9)	
H3	0.5667	0.9227	0.1475	0.056*	
C4	0.4974 (2)	0.7938 (2)	0.11289 (15)	0.0389 (8)	
C5	0.4049 (2)	0.7492 (2)	0.09027 (14)	0.0363 (8)	
C6	0.3216 (2)	0.8033 (2)	0.08845 (15)	0.0412 (8)	
C7	0.5861 (2)	0.7338 (3)	0.11817 (16)	0.0454 (9)	
C8	0.7599 (3)	0.7343 (3)	0.1475 (2)	0.0779 (13)	
H8A	0.7660	0.6831	0.1804	0.117*	
H8B	0.8137	0.7798	0.1629	0.117*	
H8C	0.7603	0.7049	0.1059	0.117*	
C9	0.3917 (2)	0.6403 (3)	0.07216 (18)	0.0431 (8)	
C10	0.3676 (3)	0.5227 (3)	−0.0148 (2)	0.0868 (14)	
H10A	0.4082	0.4780	0.0179	0.130*	
H10B	0.3811	0.5153	−0.0564	0.130*	
H10C	0.2999	0.5076	−0.0215	0.130*	
C11	0.2216 (2)	0.7587 (2)	0.06406 (16)	0.0400 (8)	
C12	0.1815 (3)	0.7259 (3)	−0.00211 (16)	0.0503 (9)	
H12	0.2171	0.7329	−0.0319	0.060*	
C13	0.0899 (2)	0.6834 (3)	−0.02369 (17)	0.0506 (9)	
H13	0.0647	0.6613	−0.0678	0.061*	
C14	0.0347 (2)	0.6729 (2)	0.01933 (16)	0.0431 (8)	
C15	0.0746 (2)	0.7055 (2)	0.08603 (16)	0.0408 (8)	
C16	0.1658 (2)	0.7487 (2)	0.10680 (15)	0.0421 (8)	
H16	0.1907	0.7718	0.1506	0.051*	
C17	0.0229 (3)	0.6902 (3)	0.13731 (18)	0.0508 (9)	
C18	−0.0328 (3)	0.5667 (3)	0.1968 (2)	0.0904 (15)	
H18A	−0.0385	0.6229	0.2234	0.136*	
H18B	0.0005	0.5138	0.2257	0.136*	
H18C	−0.0969	0.5448	0.1704	0.136*	
C19	−0.0661 (3)	0.6322 (3)	−0.00428 (19)	0.0522 (9)	
C20	−0.1833 (3)	0.5486 (3)	−0.0923 (2)	0.0846 (14)	
H20A	−0.1894	0.4880	−0.0698	0.127*	
H20B	−0.1953	0.5356	−0.1391	0.127*	
H20C	−0.2301	0.5961	−0.0870	0.127*	
O9	0.90819 (19)	0.3219 (2)	0.20321 (13)	0.0730 (8)	
O10	0.8394 (2)	0.1751 (2)	0.19739 (18)	0.0968 (11)	
O11	0.62367 (18)	0.09734 (17)	0.17382 (12)	0.0558 (7)	
O12	0.6628 (2)	0.15331 (17)	0.08491 (12)	0.0649 (7)	
O13	0.2462 (2)	0.0867 (2)	0.19228 (14)	0.0765 (8)	
O14	0.2231 (2)	0.2456 (2)	0.21508 (15)	0.0870 (10)	
O15	0.1182 (2)	0.1385 (2)	0.06290 (15)	0.0838 (9)	
O16	0.17875 (19)	0.0866 (2)	−0.01709 (14)	0.0790 (8)	
C21	0.5672 (3)	0.4153 (3)	0.18722 (18)	0.0582 (10)	
H21	0.5100	0.4484	0.1863	0.070*	
C22	0.6571 (3)	0.4616 (3)	0.21457 (18)	0.0625 (11)	
H22	0.6598	0.5251	0.2325	0.075*	
C23	0.7419 (3)	0.4149 (3)	0.21548 (17)	0.0542 (10)	
H23	0.8014	0.4478	0.2328	0.065*	
C24	0.7401 (2)	0.3184 (2)	0.19076 (16)	0.0446 (9)	
C25	0.6495 (2)	0.2707 (2)	0.16425 (15)	0.0416 (8)	
C26	0.5620 (2)	0.3192 (2)	0.16103 (15)	0.0442 (8)	
C27	0.8319 (3)	0.2630 (3)	0.19680 (17)	0.0506 (9)	
C28	1.0022 (3)	0.2714 (4)	0.2128 (2)	0.0868 (14)	
H28A	1.0181	0.2315	0.2527	0.130*	
H28B	1.0526	0.3201	0.2172	0.130*	
H28C	0.9973	0.2297	0.1748	0.130*	
C29	0.6447 (2)	0.1636 (2)	0.14292 (17)	0.0413 (8)	
C30	0.6633 (4)	0.0514 (3)	0.0632 (2)	0.1001 (17)	
H30A	0.7030	0.0119	0.0997	0.150*	
H30B	0.6896	0.0486	0.0265	0.150*	
H30C	0.5975	0.0261	0.0488	0.150*	
C31	0.4657 (2)	0.2706 (2)	0.13009 (17)	0.0448 (8)	
C32	0.4016 (3)	0.2537 (3)	0.16732 (17)	0.0513 (9)	
H32	0.4192	0.2751	0.2116	0.062*	
C33	0.3130 (3)	0.2060 (3)	0.13961 (17)	0.0478 (9)	
C34	0.2835 (2)	0.1771 (3)	0.07211 (17)	0.0484 (9)	
C35	0.3463 (3)	0.1945 (3)	0.03489 (17)	0.0548 (10)	
H35	0.3278	0.1752	−0.0099	0.066*	
C36	0.4362 (3)	0.2404 (3)	0.06343 (17)	0.0546 (10)	
H36	0.4772	0.2510	0.0377	0.066*	
C37	0.2537 (3)	0.1852 (3)	0.1855 (2)	0.0604 (10)	
C38	0.1830 (4)	0.0548 (4)	0.2310 (3)	0.1100 (18)	
H38A	0.1968	0.0941	0.2711	0.165*	
H38B	0.1951	−0.0140	0.2429	0.165*	
H38C	0.1156	0.0634	0.2045	0.165*	
C39	0.1845 (3)	0.1319 (3)	0.0402 (2)	0.0579 (10)	
C40	0.0844 (3)	0.0424 (4)	−0.0540 (2)	0.1078 (17)	
H40A	0.0676	−0.0072	−0.0267	0.162*	
H40B	0.0886	0.0122	−0.0944	0.162*	
H40C	0.0349	0.0931	−0.0652	0.162*	

### Atomic displacement parameters (Å^2^)

**Table:**

	*U*^11^	*U*^22^	*U*^33^	*U*^12^	*U*^13^	*U*^23^
O1	0.0541 (17)	0.0528 (18)	0.107 (2)	0.0050 (14)	0.0223 (15)	−0.0148 (16)
O2	0.0398 (16)	0.0647 (18)	0.0844 (19)	−0.0051 (14)	0.0202 (14)	−0.0119 (14)
O3	0.0691 (18)	0.0385 (15)	0.0755 (18)	−0.0081 (13)	0.0263 (15)	0.0061 (13)
O4	0.0716 (18)	0.0473 (15)	0.0569 (15)	−0.0036 (13)	0.0221 (13)	−0.0166 (12)
O5	0.0744 (19)	0.0595 (18)	0.0815 (19)	0.0036 (14)	0.0446 (15)	0.0158 (14)
O6	0.079 (2)	0.0711 (19)	0.0828 (19)	0.0027 (16)	0.0499 (17)	−0.0061 (16)
O7	0.0496 (17)	0.117 (3)	0.086 (2)	−0.0181 (17)	0.0287 (16)	−0.0200 (18)
O8	0.0518 (17)	0.0757 (19)	0.0743 (19)	−0.0166 (14)	0.0123 (14)	−0.0197 (15)
C1	0.048 (2)	0.037 (2)	0.065 (2)	0.0078 (18)	0.0200 (19)	−0.0003 (18)
C2	0.052 (2)	0.035 (2)	0.065 (2)	−0.0052 (19)	0.0175 (19)	−0.0066 (17)
C3	0.041 (2)	0.045 (2)	0.050 (2)	−0.0102 (18)	0.0102 (17)	−0.0029 (17)
C4	0.041 (2)	0.037 (2)	0.0397 (18)	−0.0047 (17)	0.0139 (16)	−0.0010 (15)
C5	0.040 (2)	0.0340 (19)	0.0376 (17)	−0.0015 (16)	0.0159 (15)	−0.0031 (14)
C6	0.043 (2)	0.040 (2)	0.0428 (19)	−0.0040 (17)	0.0162 (16)	−0.0021 (16)
C7	0.039 (2)	0.051 (2)	0.046 (2)	−0.006 (2)	0.0132 (17)	−0.0085 (18)
C8	0.042 (2)	0.097 (3)	0.097 (3)	0.003 (2)	0.025 (2)	−0.009 (3)
C9	0.036 (2)	0.038 (2)	0.053 (2)	−0.0046 (16)	0.0122 (17)	−0.0080 (18)
C10	0.108 (4)	0.054 (3)	0.099 (3)	−0.012 (2)	0.032 (3)	−0.040 (2)
C11	0.0333 (19)	0.041 (2)	0.046 (2)	0.0034 (16)	0.0129 (16)	0.0048 (16)
C12	0.049 (2)	0.063 (2)	0.045 (2)	−0.005 (2)	0.0236 (18)	0.0031 (18)
C13	0.044 (2)	0.063 (2)	0.043 (2)	−0.0078 (19)	0.0101 (17)	−0.0032 (18)
C14	0.038 (2)	0.043 (2)	0.049 (2)	0.0002 (17)	0.0156 (17)	0.0048 (16)
C15	0.035 (2)	0.043 (2)	0.047 (2)	0.0055 (17)	0.0177 (16)	0.0065 (16)
C16	0.042 (2)	0.047 (2)	0.0380 (18)	0.0012 (17)	0.0128 (16)	−0.0007 (16)
C17	0.043 (2)	0.056 (3)	0.055 (2)	−0.0043 (19)	0.0188 (18)	0.006 (2)
C18	0.099 (4)	0.091 (3)	0.102 (3)	−0.012 (3)	0.061 (3)	0.027 (3)
C19	0.041 (2)	0.057 (2)	0.056 (2)	−0.0014 (19)	0.0112 (19)	0.004 (2)
C20	0.057 (3)	0.083 (3)	0.097 (3)	−0.016 (2)	−0.001 (2)	−0.015 (3)
O9	0.0493 (17)	0.0671 (18)	0.105 (2)	−0.0108 (15)	0.0274 (15)	−0.0144 (16)
O10	0.068 (2)	0.0443 (19)	0.179 (3)	0.0064 (15)	0.040 (2)	0.0258 (19)
O11	0.0757 (18)	0.0373 (14)	0.0639 (16)	−0.0082 (13)	0.0355 (14)	0.0004 (12)
O12	0.107 (2)	0.0389 (15)	0.0671 (17)	0.0001 (14)	0.0528 (16)	−0.0036 (12)
O13	0.083 (2)	0.073 (2)	0.086 (2)	0.0099 (16)	0.0456 (17)	0.0200 (16)
O14	0.089 (2)	0.089 (2)	0.106 (2)	0.0118 (18)	0.064 (2)	−0.0156 (19)
O15	0.0512 (18)	0.110 (3)	0.092 (2)	−0.0072 (17)	0.0262 (16)	0.0036 (18)
O16	0.0606 (19)	0.092 (2)	0.076 (2)	−0.0128 (16)	0.0102 (15)	−0.0203 (17)
C21	0.063 (3)	0.045 (2)	0.064 (2)	0.016 (2)	0.017 (2)	−0.0009 (19)
C22	0.078 (3)	0.037 (2)	0.066 (3)	0.003 (2)	0.014 (2)	−0.0107 (18)
C23	0.063 (3)	0.040 (2)	0.054 (2)	−0.014 (2)	0.0111 (19)	0.0025 (18)
C24	0.051 (2)	0.035 (2)	0.047 (2)	−0.0040 (18)	0.0135 (17)	0.0053 (16)
C25	0.051 (2)	0.0314 (19)	0.0431 (19)	0.0020 (17)	0.0155 (17)	0.0035 (15)
C26	0.047 (2)	0.040 (2)	0.046 (2)	0.0035 (18)	0.0131 (17)	0.0028 (16)
C27	0.045 (2)	0.052 (3)	0.052 (2)	−0.003 (2)	0.0130 (18)	0.0053 (18)
C28	0.052 (3)	0.110 (4)	0.104 (4)	−0.005 (3)	0.033 (3)	−0.019 (3)
C29	0.045 (2)	0.038 (2)	0.044 (2)	−0.0016 (17)	0.0178 (17)	−0.0008 (17)
C30	0.186 (6)	0.047 (3)	0.099 (3)	−0.003 (3)	0.090 (4)	−0.020 (2)
C31	0.043 (2)	0.040 (2)	0.050 (2)	0.0068 (17)	0.0127 (18)	0.0003 (16)
C32	0.053 (2)	0.053 (2)	0.048 (2)	0.018 (2)	0.0161 (19)	0.0037 (18)
C33	0.045 (2)	0.046 (2)	0.053 (2)	0.0133 (18)	0.0156 (18)	0.0049 (17)
C34	0.037 (2)	0.050 (2)	0.058 (2)	0.0106 (18)	0.0142 (19)	0.0061 (18)
C35	0.053 (2)	0.067 (3)	0.040 (2)	0.006 (2)	0.0094 (19)	0.0007 (18)
C36	0.053 (2)	0.062 (2)	0.050 (2)	−0.004 (2)	0.0187 (19)	−0.0010 (19)
C37	0.049 (2)	0.070 (3)	0.064 (3)	0.009 (2)	0.021 (2)	0.006 (2)
C38	0.131 (4)	0.105 (4)	0.122 (4)	−0.001 (3)	0.080 (4)	0.032 (3)
C39	0.051 (3)	0.058 (3)	0.060 (3)	0.008 (2)	0.011 (2)	0.012 (2)
C40	0.078 (4)	0.123 (4)	0.106 (4)	−0.041 (3)	0.004 (3)	−0.026 (3)

### Geometric parameters (Å, °)

**Table:**

O1—C7	1.188 (4)	O9—C27	1.326 (4)
O2—C7	1.331 (4)	O9—C28	1.469 (4)
O2—C8	1.465 (4)	O10—C27	1.188 (4)
O3—C9	1.205 (4)	O11—C29	1.197 (4)
O4—C9	1.328 (4)	O12—C29	1.331 (4)
O4—C10	1.457 (4)	O12—C30	1.446 (4)
O5—C17	1.323 (4)	O13—C37	1.340 (4)
O5—C18	1.452 (4)	O13—C38	1.465 (4)
O6—C17	1.206 (4)	O14—C37	1.190 (4)
O7—C19	1.209 (4)	O15—C39	1.198 (4)
O8—C19	1.320 (4)	O16—C39	1.328 (4)
O8—C20	1.454 (4)	O16—C40	1.461 (5)
C1—C2	1.375 (5)	C21—C22	1.386 (5)
C1—C6	1.406 (4)	C21—C26	1.399 (4)
C1—H1	0.9300	C21—H21	0.9300
C2—C3	1.379 (4)	C22—C23	1.367 (5)
C2—H2	0.9300	C22—H22	0.9300
C3—C4	1.386 (4)	C23—C24	1.396 (4)
C3—H3	0.9300	C23—H23	0.9300
C4—C5	1.399 (4)	C24—C25	1.401 (4)
C4—C7	1.485 (4)	C24—C27	1.487 (5)
C5—C6	1.394 (4)	C25—C26	1.401 (4)
C5—C9	1.510 (4)	C25—C29	1.504 (4)
C6—C11	1.492 (4)	C26—C31	1.484 (5)
C8—H8A	0.9600	C28—H28A	0.9600
C8—H8B	0.9600	C28—H28B	0.9600
C8—H8C	0.9600	C28—H28C	0.9600
C10—H10A	0.9600	C30—H30A	0.9600
C10—H10B	0.9600	C30—H30B	0.9600
C10—H10C	0.9600	C30—H30C	0.9600
C11—C16	1.389 (4)	C31—C36	1.390 (4)
C11—C12	1.398 (4)	C31—C32	1.404 (4)
C12—C13	1.376 (4)	C32—C33	1.381 (5)
C12—H12	0.9300	C32—H32	0.9300
C13—C14	1.387 (4)	C33—C34	1.401 (5)
C13—H13	0.9300	C33—C37	1.502 (5)
C14—C15	1.406 (4)	C34—C35	1.391 (5)
C14—C19	1.481 (5)	C34—C39	1.497 (5)
C15—C16	1.373 (4)	C35—C36	1.386 (5)
C15—C17	1.506 (4)	C35—H35	0.9300
C16—H16	0.9300	C36—H36	0.9300
C18—H18A	0.9600	C38—H38A	0.9600
C18—H18B	0.9600	C38—H38B	0.9600
C18—H18C	0.9600	C38—H38C	0.9600
C20—H20A	0.9600	C40—H40A	0.9600
C20—H20B	0.9600	C40—H40B	0.9600
C20—H20C	0.9600	C40—H40C	0.9600
			
C7—O2—C8	116.6 (3)	C27—O9—C28	115.8 (3)
C9—O4—C10	115.6 (3)	C29—O12—C30	114.2 (3)
C17—O5—C18	117.0 (3)	C37—O13—C38	115.8 (3)
C19—O8—C20	116.2 (3)	C39—O16—C40	116.7 (3)
C2—C1—C6	120.1 (3)	C22—C21—C26	120.3 (4)
C2—C1—H1	119.9	C22—C21—H21	119.9
C6—C1—H1	119.9	C26—C21—H21	119.9
C1—C2—C3	120.7 (3)	C23—C22—C21	120.8 (4)
C1—C2—H2	119.6	C23—C22—H22	119.6
C3—C2—H2	119.6	C21—C22—H22	119.6
C2—C3—C4	120.3 (3)	C22—C23—C24	120.7 (4)
C2—C3—H3	119.9	C22—C23—H23	119.6
C4—C3—H3	119.9	C24—C23—H23	119.6
C3—C4—C5	119.6 (3)	C23—C24—C25	118.7 (3)
C3—C4—C7	120.7 (3)	C23—C24—C27	121.5 (3)
C5—C4—C7	119.6 (3)	C25—C24—C27	119.6 (3)
C6—C5—C4	120.2 (3)	C24—C25—C26	121.0 (3)
C6—C5—C9	117.4 (3)	C24—C25—C29	120.4 (3)
C4—C5—C9	122.2 (3)	C26—C25—C29	118.4 (3)
C5—C6—C1	119.1 (3)	C21—C26—C25	118.5 (3)
C5—C6—C11	121.8 (3)	C21—C26—C31	120.4 (3)
C1—C6—C11	119.1 (3)	C25—C26—C31	121.1 (3)
O1—C7—O2	122.8 (3)	O10—C27—O9	121.6 (4)
O1—C7—C4	124.8 (3)	O10—C27—C24	125.2 (4)
O2—C7—C4	112.4 (3)	O9—C27—C24	113.3 (3)
O2—C8—H8A	109.5	O9—C28—H28A	109.5
O2—C8—H8B	109.5	O9—C28—H28B	109.5
H8A—C8—H8B	109.5	H28A—C28—H28B	109.5
O2—C8—H8C	109.5	O9—C28—H28C	109.5
H8A—C8—H8C	109.5	H28A—C28—H28C	109.5
H8B—C8—H8C	109.5	H28B—C28—H28C	109.5
O3—C9—O4	126.2 (3)	O11—C29—O12	125.2 (3)
O3—C9—C5	122.1 (3)	O11—C29—C25	123.2 (3)
O4—C9—C5	111.6 (3)	O12—C29—C25	111.6 (3)
O4—C10—H10A	109.5	O12—C30—H30A	109.5
O4—C10—H10B	109.5	O12—C30—H30B	109.5
H10A—C10—H10B	109.5	H30A—C30—H30B	109.5
O4—C10—H10C	109.5	O12—C30—H30C	109.5
H10A—C10—H10C	109.5	H30A—C30—H30C	109.5
H10B—C10—H10C	109.5	H30B—C30—H30C	109.5
C16—C11—C12	118.1 (3)	C36—C31—C32	118.0 (3)
C16—C11—C6	120.6 (3)	C36—C31—C26	121.1 (3)
C12—C11—C6	121.2 (3)	C32—C31—C26	121.0 (3)
C13—C12—C11	120.7 (3)	C33—C32—C31	121.5 (3)
C13—C12—H12	119.7	C33—C32—H32	119.3
C11—C12—H12	119.7	C31—C32—H32	119.3
C12—C13—C14	121.0 (3)	C32—C33—C34	120.0 (3)
C12—C13—H13	119.5	C32—C33—C37	116.9 (3)
C14—C13—H13	119.5	C34—C33—C37	123.1 (3)
C13—C14—C15	118.6 (3)	C35—C34—C33	118.6 (3)
C13—C14—C19	121.5 (3)	C35—C34—C39	120.8 (3)
C15—C14—C19	119.8 (3)	C33—C34—C39	120.5 (3)
C16—C15—C14	119.8 (3)	C36—C35—C34	121.1 (3)
C16—C15—C17	118.0 (3)	C36—C35—H35	119.5
C14—C15—C17	122.1 (3)	C34—C35—H35	119.5
C15—C16—C11	121.7 (3)	C35—C36—C31	120.8 (3)
C15—C16—H16	119.1	C35—C36—H36	119.6
C11—C16—H16	119.1	C31—C36—H36	119.6
O6—C17—O5	124.2 (3)	O14—C37—O13	124.3 (4)
O6—C17—C15	125.3 (3)	O14—C37—C33	126.0 (4)
O5—C17—C15	110.3 (3)	O13—C37—C33	109.5 (3)
O5—C18—H18A	109.5	O13—C38—H38A	109.5
O5—C18—H18B	109.5	O13—C38—H38B	109.5
H18A—C18—H18B	109.5	H38A—C38—H38B	109.5
O5—C18—H18C	109.5	O13—C38—H38C	109.5
H18A—C18—H18C	109.5	H38A—C38—H38C	109.5
H18B—C18—H18C	109.5	H38B—C38—H38C	109.5
O7—C19—O8	124.1 (3)	O15—C39—O16	124.4 (4)
O7—C19—C14	124.3 (4)	O15—C39—C34	123.8 (4)
O8—C19—C14	111.6 (3)	O16—C39—C34	111.8 (3)
O8—C20—H20A	109.5	O16—C40—H40A	109.5
O8—C20—H20B	109.5	O16—C40—H40B	109.5
H20A—C20—H20B	109.5	H40A—C40—H40B	109.5
O8—C20—H20C	109.5	O16—C40—H40C	109.5
H20A—C20—H20C	109.5	H40A—C40—H40C	109.5
H20B—C20—H20C	109.5	H40B—C40—H40C	109.5
			
C6—C1—C2—C3	−0.7 (5)	C26—C21—C22—C23	1.1 (6)
C1—C2—C3—C4	−0.8 (5)	C21—C22—C23—C24	−2.0 (5)
C2—C3—C4—C5	1.3 (5)	C22—C23—C24—C25	0.7 (5)
C2—C3—C4—C7	−175.6 (3)	C22—C23—C24—C27	−174.5 (3)
C3—C4—C5—C6	−0.3 (4)	C23—C24—C25—C26	1.5 (5)
C7—C4—C5—C6	176.6 (3)	C27—C24—C25—C26	176.7 (3)
C3—C4—C5—C9	−175.5 (3)	C23—C24—C25—C29	−174.4 (3)
C7—C4—C5—C9	1.4 (4)	C27—C24—C25—C29	0.9 (5)
C4—C5—C6—C1	−1.2 (4)	C22—C21—C26—C25	1.1 (5)
C9—C5—C6—C1	174.3 (3)	C22—C21—C26—C31	−178.4 (3)
C4—C5—C6—C11	179.4 (3)	C24—C25—C26—C21	−2.3 (5)
C9—C5—C6—C11	−5.1 (4)	C29—C25—C26—C21	173.5 (3)
C2—C1—C6—C5	1.7 (5)	C24—C25—C26—C31	177.1 (3)
C2—C1—C6—C11	−178.9 (3)	C29—C25—C26—C31	−7.0 (4)
C8—O2—C7—O1	−3.9 (5)	C28—O9—C27—O10	−1.6 (5)
C8—O2—C7—C4	176.6 (3)	C28—O9—C27—C24	177.0 (3)
C3—C4—C7—O1	174.1 (3)	C23—C24—C27—O10	154.8 (4)
C5—C4—C7—O1	−2.7 (5)	C25—C24—C27—O10	−20.3 (5)
C3—C4—C7—O2	−6.4 (4)	C23—C24—C27—O9	−23.7 (4)
C5—C4—C7—O2	176.8 (3)	C25—C24—C27—O9	161.1 (3)
C10—O4—C9—O3	−0.5 (5)	C30—O12—C29—O11	−5.0 (5)
C10—O4—C9—C5	−175.4 (3)	C30—O12—C29—C25	177.5 (3)
C6—C5—C9—O3	−76.6 (4)	C24—C25—C29—O11	107.1 (4)
C4—C5—C9—O3	98.8 (4)	C26—C25—C29—O11	−68.8 (4)
C6—C5—C9—O4	98.6 (3)	C24—C25—C29—O12	−75.4 (4)
C4—C5—C9—O4	−86.0 (4)	C26—C25—C29—O12	108.7 (3)
C5—C6—C11—C16	117.0 (3)	C21—C26—C31—C36	119.8 (4)
C1—C6—C11—C16	−62.4 (4)	C25—C26—C31—C36	−59.6 (4)
C5—C6—C11—C12	−63.0 (4)	C21—C26—C31—C32	−60.2 (4)
C1—C6—C11—C12	117.6 (4)	C25—C26—C31—C32	120.3 (4)
C16—C11—C12—C13	−1.1 (5)	C36—C31—C32—C33	1.8 (5)
C6—C11—C12—C13	178.9 (3)	C26—C31—C32—C33	−178.1 (3)
C11—C12—C13—C14	0.7 (5)	C31—C32—C33—C34	−2.7 (5)
C12—C13—C14—C15	−0.8 (5)	C31—C32—C33—C37	175.6 (3)
C12—C13—C14—C19	176.7 (3)	C32—C33—C34—C35	2.0 (5)
C13—C14—C15—C16	1.4 (5)	C37—C33—C34—C35	−176.1 (3)
C19—C14—C15—C16	−176.1 (3)	C32—C33—C34—C39	−175.6 (3)
C13—C14—C15—C17	−175.5 (3)	C37—C33—C34—C39	6.2 (5)
C19—C14—C15—C17	7.0 (5)	C33—C34—C35—C36	−0.5 (5)
C14—C15—C16—C11	−1.9 (5)	C39—C34—C35—C36	177.1 (3)
C17—C15—C16—C11	175.1 (3)	C34—C35—C36—C31	−0.3 (6)
C12—C11—C16—C15	1.7 (5)	C32—C31—C36—C35	−0.3 (5)
C6—C11—C16—C15	−178.3 (3)	C26—C31—C36—C35	179.6 (3)
C18—O5—C17—O6	10.4 (5)	C38—O13—C37—O14	9.6 (6)
C18—O5—C17—C15	−174.3 (3)	C38—O13—C37—C33	−174.8 (3)
C16—C15—C17—O6	68.0 (5)	C32—C33—C37—O14	60.3 (5)
C14—C15—C17—O6	−115.2 (4)	C34—C33—C37—O14	−121.5 (4)
C16—C15—C17—O5	−107.3 (4)	C32—C33—C37—O13	−115.2 (4)
C14—C15—C17—O5	69.6 (4)	C34—C33—C37—O13	63.0 (4)
C20—O8—C19—O7	0.3 (5)	C40—O16—C39—O15	0.1 (6)
C20—O8—C19—C14	−177.9 (3)	C40—O16—C39—C34	−178.0 (3)
C13—C14—C19—O7	−163.5 (4)	C35—C34—C39—O15	−159.6 (4)
C15—C14—C19—O7	13.9 (5)	C33—C34—C39—O15	17.9 (6)
C13—C14—C19—O8	14.6 (5)	C35—C34—C39—O16	18.5 (5)
C15—C14—C19—O8	−168.0 (3)	C33—C34—C39—O16	−163.9 (3)

## References

[bb1] Ding, M. X., Wang, Z. G., Yang, Z. H. & Zhang, J. (1992). US Patent 5081281.

[bb2] Ermer, O. (1981). *Helv. Chim. Acta*, **64**, 1902–1909.

[bb3] Farrugia, L. J. (1997). *J. Appl. Cryst.* **30**, 565.

[bb4] Gabe, E. J., Le Page, Y., Charland, J.-P., Lee, F. L. & White, P. S. (1989). *J. Appl. Cryst.* **22**, 384–387.

[bb5] Gabe, E. J. & White, P. S. (1993). *Am. Crystallogr. Assoc. Pittsburgh Meet.* Abstract PA104.

[bb6] Ghosh, M. K. & Mittal, K. L. (1996). *Polyimides, Fundamentals and Applications* New York: Dekker.

[bb7] Jiang, Y., Men, J., Liu, C.-Y., Zhang, Y. & Gao, G.-W. (2008). *Acta Cryst.* E**64**, o846. 10.1107/S1600536808009689 PMC2961247 21202334

[bb8] Rozhanskii, I., Okuyama, K. & Goto, K. (2000). *Polymer*, **41**, 7057–7065.

[bb9] Sheldrick, G. M. (2008). *Acta Cryst.* A**64**, 112–122. 10.1107/S0108767307043930 18156677

